# Suppression of LPS-induced inflammatory responses in macrophages infected with *Leishmania*

**DOI:** 10.1186/1476-9255-7-8

**Published:** 2010-02-02

**Authors:** Nicholas J Lapara, Ben L Kelly

**Affiliations:** 1 Department of Microbiology Immunology and Parasitology, LSU Health Sciences Center, 1901 Perdido Street, New Orleans, LA 70112, USA

## Abstract

**Background:**

Chronic inflammation activated by macrophage innate pathogen recognition receptors such as TLR4 can lead to a range of inflammatory diseases, including atherosclerosis, Crohn's disease, arthritis and cancer. Unlike many microbes, the kinetoplastid protozoan pathogen *Leishmania *has been shown to avoid and even actively suppress host inflammatory cytokine responses, such as LPS-induced IL-12 production. The nature and scope of *Leishmania*-mediated inflammatory cytokine suppression, however, is not well characterized. Advancing our knowledge of such microbe-mediated cytokine suppression may provide new avenues for therapeutic intervention in inflammatory disease.

**Methods:**

We explored the kinetics of a range of cytokine and chemokine responses in primary murine macrophages stimulated with LPS in the presence versus absence of two clinically distinct species of *Leishmania *using sensitive multiplex cytokine analyses. To confirm that these effects were parasite-specific, we compared the effects of *Leishmania *uptake on LPS-induced cytokine expression with uptake of inert latex beads.

**Results:**

Whilst *Leishmania *uptake alone did not induce significant levels of any cytokine analysed in this study, *Leishmania *uptake in the presence of LPS caused parasite-specific suppression of certain LPS-induced pro-inflammatory cytokines, including IL-12, IL-17 and IL-6. Interestingly, *L. amazonensis *was generally more suppressive than *L. major*. We also found that other LPS-induced proinflammatory cytokines, such as IL-1α, TNF-α and the chemokines MIP-1α and MCP-1 and also the anti-inflammatory cytokine IL-10, were augmented during *Leishmania *uptake, in a parasite-specific manner.

**Conclusions:**

During uptake by macrophages, *Leishmania *evades the activation of a broad range of cytokines and chemokines. Further, in the presence of a strong inflammatory stimulus, *Leishmania *suppresses certain proinflammatory cytokine responses in a parasite-specific manner, however it augments the production of other proinflammatory cytokines. Our findings highlight the complexity of inflammatory cytokine signalling regulation in the context of the macrophage and *Leishmania *interaction and confirm the utility of the *Leishmania*/macrophage infection model as an experimental system for further studies of inflammatory regulation. Such studies may advance the development of therapies against inflammatory disease.

## Background

Inflammatory diseases such as atherosclerosis are often caused by chronic inflammation and encompass a large spectrum of diseases, including atherosclerosis, Crohn's disease, arthritis and cancer [1-6]. Persistence of, or repeated exposure to certain microbial pathogens, leads to chronically elevated levels of several etiologic inflammatory mediators, including the cytokines IL-12, tumor necrosis factor alpha (TNF-α), interferon-gamma (IFN-γ), IL-6 and IL-17 [1,7], that are considered to contribute to the onset of inflammatory diseases. Production of these cytokines typically ensues via host cell signalling cascades following the engagement of innate pathogen-associated molecular pattern (PAMP) receptors including the Toll-like receptors (TLRs) expressed primarily by cells of the innate immune compartment, by pathogen-specific ligands, such as bacterial lipopolysaccharide (LPS) [8]. Importantly, recent studies with gene-knockout mice and also human TLR polymorphisms have confirmed that TLR signaling in response to pathogen contact can also contribute to the development of atherosclerosis [9,10]. The mechanisms that underlie inflammatory cytokine induction following engagement of the TLR receptors have been well studied for a number of cytokines and commonly result in nuclear translocation of nuclear factor kappa-light-chain-enhancer of activated B cells (NF-κB), a Rel-family transcription factor, followed by its binding to DNA sequences associated with the promoters of their target genes such as IL-12 [11]. In addition to NF-κB activation, TLR signaling may also activate mitogen activated protein (MAP) kinase pathways that lead to activation of the transcription factor activating protein 1 (AP-1) and interferon regulatory factor (IRF) signalling [1].

In contrast, whilst macrophage IL-17 production has been demonstrated to be important in allergic inflammation [7], pathways that culminate in the regulation of this proinflammatory cytokine in macrophages, however, have not yet been characterized.

Unlike most microbial pathogens, insect stages (promastigotes) of the protozoan trypanosomatid parasite *Leishmania *enter macrophage host cells in a way that eludes immediate "classical" proinflammatory activation. Furthermore, a number of studies have shown that upon LPS stimulation of macrophages, IL-12 is actively suppressed by *Leishmania *[12,13]. *Leishmania major-*infected C57/BL6 mice, also show an initial "silent" phase of parasite replication that persists in the dermis for up to 5 weeks [14] prior to disease resolution, indicating parasite-mediated immune evasion occurs *in vivo*. Experimental infections with *L. amazonensis *also show early impairment of inflammatory responses [15]. Importantly, microarray experiments also showed that infection of the human monocyte line, THP-1, with *Leishmania *suppressed the IFNγ-induced expression of many host genes [16].

Cameron *et al*., [12] showed that the suppression of LPS-induced IL-12 by *L. mexicana *correlated with degradation of the innate immune signaling MAP kinases JNK and ERK, and also components of the NF-κB signaling pathway, indicating that *Leishmania *may promote a generalized abrogation of the inflammatory response.

Additional mechanisms that have been proposed for *Leishmania*'s ability to suppress inflammatory activation include the engagement of suppression-associated macrophage surface receptors such as complement receptor 3 (CR3) and the elaboration of suppressive cytokines such as IL-10 [17,18]. Indeed, studies of macrophages from CR3-deficient mice have confirmed that CR3 engagement is involved in IL-12 suppression during *Leishmania *infection even in the presence of IFN-γ stimulation [17,19].

Although some progress toward understanding how *Leishmania *suppresses macrophage inflammatory responses has been made, our knowledge of the extent to which *Leishmania *modulates macrophage cytokine responses and the underlying molecular mechanisms involved, remains limited.

To further our understanding of host inflammatory responses modulated by *Leishmania*, we have explored the inflammation-suppressive effects of both *L. major *and *L. amazonensis *in the context of macrophage infection during TLR4 stimulation, upon a broader range of cytokines than previously studied. These parasites represent two related but distinct microbes responsible for clinically distinct forms of leishmaniasis. Specifically, we studied the modulatory effects of *L. major *and *L. amazonensis *upon the proinflammatory cytokines IL-17, IL-1α and TNF-α, the primary Th1-inducing proinflammatory cytokine, IL-12, and the Th2-associated cytokines IL-4,-13, -6,-10 and IL-3 [20]. We also assayed for the macrophage inflammatory chemokines macrophage inflammatory protein 1α (MIP-1α) and monocyte chemotactic protein-1 (MCP-1). We find that, although LPS-induced IL-17 and IL-12 are repressed by both *Leishmania *species, LPS-induced TNF-α and IL-1α responses are enhanced. In addition, we determined that both *Leishmania *species suppress LPS-induced IL-6, -13 and -3. Furthermore, *L. amazonensis *also suppressed LPS-induced IL-4 and IL-10 whereas both *Leishmania *species augmented LPS-induced MIP-1α and MCP-1 production. Our findings suggest, at least in the context of TLR4 stimulation, that *Leishmania *promastigotes do not promote generalized proinflammatory suppression and instead appear to target specific cytokine signalling pathways downstream of the TLR4 receptor, to selectively modulate cytokine and chemokine production during macrophage parasitization.

## Methods

### Isolation and Culture of Peritoneal Macrophages

Peritoneal cells were isolated by lavage from C57/BL6 mice that were purchased from The Jackson Laboratory essentially as described previously [21], with the exception that RPMI-1640/10% FBS was used for peritoneal lavage.

The peritoneal cells were stained with macrophage markers F4/80, CD14, CD11b and CD 205 then analysed by flow cytometry to confirm their macrophage phenotype and adjusted to a cell density of 4 × 10^5^/ml.

### Parasites

Promastigotes of *L. major *strain WHOM/IR/-/173 and *L. amazonensis *strain IFLA/BR/67/PH8 (kindly provided by Dr David L. Sacks, NIAID, Bethesda, MD) were cultured *in vitro *at 27°C in medium 199 with 10% heat-inactivated FBS as previously described [22]. Stationary phase parasites were centrifuged at 1300 × g and resuspended in RPMI-1640 culture medium supplemented with 10% FBS prior to addition to macrophage monolayers.

### Macrophage Treatment

0.8 ml aliquots of macrophages (4 × 10^5^/ml) were incubated for 2 hr on 4-well glass chamber slides, washed to remove non-adherent cells, then co-incubated with or without LPS (100 ng/ml), followed by the addition of stationary phase promastigotes (20:1 parasite: macrophage), or 6 μm latex beads (Sigma) (20:1 bead: macrophage), as indicated in the Results. At 2, 8 or 19 hr timepoints, 200 μl of culture supernatant was removed and centrifuged to remove particulates prior to multiplex cytokine analysis.

Following removal of the 19 hr culture supernatants, the macrophages were washed twice with PBS and stained using Diff-Quik (DADE-Behring) as described previously [22], evaluated microscopically (approximately 200 fields observed) and infection/uptake rates determined to be 74%, 79% and 82% for *L. major *and *L. amazonensis*, and latex beads respectively.

### Multiplex cytokine analysis

Multiplex cytokine analyses that were performed using a Bio-Plex kit (Bio-Rad) in accordance with manufacturer's instructions and analysed using a Luminex machine (Luminex Corporation).

### ELISA assays

Duplicate culture supernatants were removed at the 19 hr timepoint and assayed for cytokine production using ELISA kits from R&D systems, in accordance with manufacturers' instructions.

## Results

### Leishmania suppresses IL-17, IL-12 and IL-3 following induction with LPS

**Figure 1:**
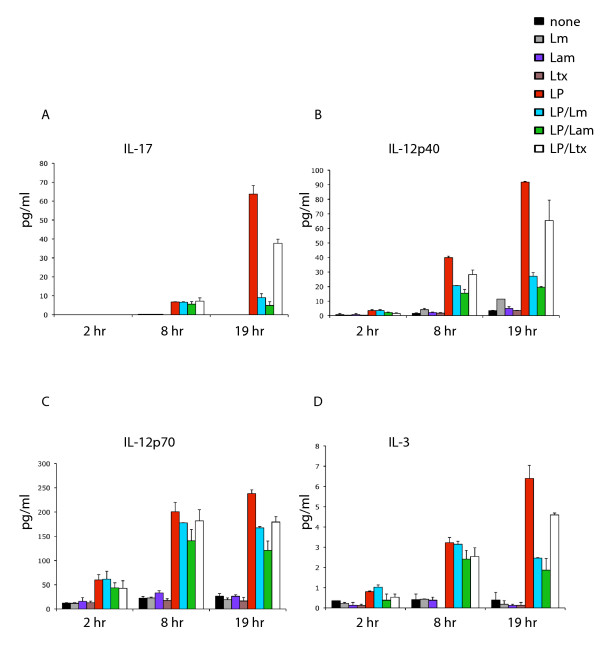
***Leishmania *suppresses IL-17, IL-12 and IL-3 following induction with LPS**. Cells were incubated with 100 ng/ml LPS with the addition of *L. major*, *L. amazonensis *or latex beads at a parasite/bead: host cell ratio of 20:1 (indicated by blue, green and open bars, respectively). At 2 hr, 8 hr and 19 hr timepoints, 200 μl of culture supernatant was removed, centrifuged to remove particulates and analysed for cytokine expression by Bio-Plex assay as described in Methods. Media only, *L. major *alone, *L. amazonensis *alone, latex beads alone and LPS alone (black, gray, purple, brown and red bars, respectively) were used as control treatments. Data represented are averages of two independent replicate experiments, with error bars as indicated.

Despite recent interest in IL-17, a key cytokine involved in a variety immune responses, including the induction of other cytokines, its production from macrophages in the context of *Leishmania *infection has not been characterized. We therefore investigated the release of IL-17 from macrophages during infection with *Leishmania *alone, or during *Leishmania *infection in the presence of LPS. Although incubation of macrophages with either *L. major *or *L. amazonensis *alone did not induce IL-17 production, nor significant levels of any other cytokine we assayed, stimulation with LPS alone caused significant IL-17 induction compared to controls, as shown in Figure 1A. However, co-incubation of the macrophages with LPS in the presence of *L. major *or *L. amazonensis *resulted in 7.1-fold and 13.1-fold suppression of IL-17, respectively, relative to LPS alone (Figure 1A). To confirm that this suppression was not merely a consequence of non-specific phagocytic uptake, we also analysed IL-17 production from LPS in the presence of 6 μm latex beads that were of comparable size to *Leishmania*. In contrast to incubation with LPS and *Leishmania*, co-incubation of LPS with latex beads caused less than two-fold suppression compared to LPS alone (Figure 1A, white bars), indicating these effects were *Leishmania*-specific. Since *L. mexicana *has previously been shown to suppress LPS-induced IL-12 production [12], we also analysed the macrophage LPS-induced IL-12 response in the presence of *L. major *and *L. amazonensis*, or latex beads as a non-specific phagocytosis control. As shown in Figure 1B, *L. major *and *L. amazonensis *suppressed peak levels of LPS-induced IL-12 p40 subunit 3.3-fold and 4.7-fold respectively, supporting previous findings [12]. In contrast, latex beads suppressed IL-12p40 production only 1.4-fold. Since it is the IL-12p70 heterodimer (IL-12p40/IL-12p35) that induces Th1 responses, we also sought to compare its regulation with IL-12p40. Although less striking than the down-regulation of IL-12 p40, *Leishmania amazonensis *mediated 2-fold down-regulation of LPS-induced IL-12p70 at 19 hrs, whereas *L. major *and latex beads, both showed modest suppression (Figure 1C). We also investigated the effects of *Leishmania *upon the induction of IL-3 since IL-3 production may promote intracellular survival because it has been associated with differentiation of monocytes into macrophages that are less responsive to IFNγ than macrophages differentiated with GM-CSF [23]. As shown in Figure 1D, whilst LPS induced IL-3 levels significantly above the control treatments, *L. major *and *L. amazonensis *suppressed LPS-induced IL-3 2.5-fold and 3.4-fold respectively. In contrast, treatment with LPS and latex beads caused 1.4-fold suppression, indicating that the IL-3 suppressive effects of *Leishmania *were specific.

### Leishmania suppresses the Th2-associated cytokines IL-4, IL-6 and IL-13 following induction with LPS

**Figure 2:**
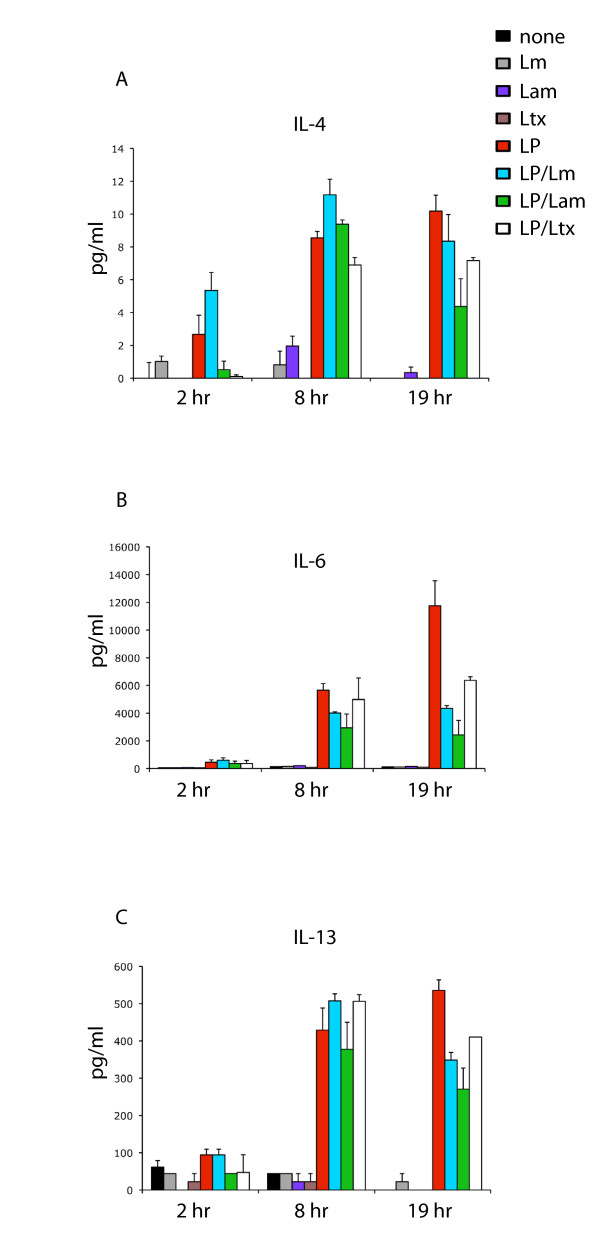
***Leishmania *suppresses the Th2-associated cytokines IL-4, IL-6 and IL-13 following induction with LPS**. Cells were incubated with LPS (100 ng/ml) followed by the addition of *L. major, L. amazonensis *or latex beads at a parasite/bead: host cell ratio of 20:1 (denoted by blue, green and open bars, respectively). At 2 hr, 8 hr and 19 hr timepoints, 200 μl of culture supernatant was removed, centrifuged and analysed for cytokine expression by Bio-Plex assay as described in Methods. Media only, *L. major *alone, *L. amazonensis *alone, latex beads alone and LPS alone (black, gray, purple, brown and red bars, respectively) were used as controls. Data represented are averages of two independent replicate experiments, with error bars as indicated.

Since susceptibility to *Leishmania *infection is generally associated with a Th2 response in infected mice, we investigated the effect of *Leishmania *on the ability of LPS to induce the Th2 cytokines IL-4, IL-6, and IL-13 from macrophages, the primary cells parasitized by *Leishmania *promastigotes. As shown in Figure 2A, whilst LPS-induced production of IL-4 was significantly upregulated by LPS alone (Figure 2A), peak production of LPS-induced IL-4 was suppressed 2.3-fold by *L. amazonensis *and 1.4-fold by latex beads, whereas negligible suppression of LPS-induced IL-4 was observed for *L. major *(1.2-fold suppression). In the presence of *L. major*, *L. amazonensis *and latex beads, LPS-induced IL-6 responses were substantially abrogated, with 2.7-fold, 4.8-fold and 1.8-fold reductions respectively. Whilst LPS induced significant levels of IL-13, *L. major*, *L. amazonensis *and latex beads showed modest suppression, down regulating LPS-induced IL-13 1.5 fold, 2-fold and 1.3-fold respectively (Figure 2B).

### Leishmania augments release of IL-1α, TNFα and IL-10 following LPS induction

**Figure 3:**
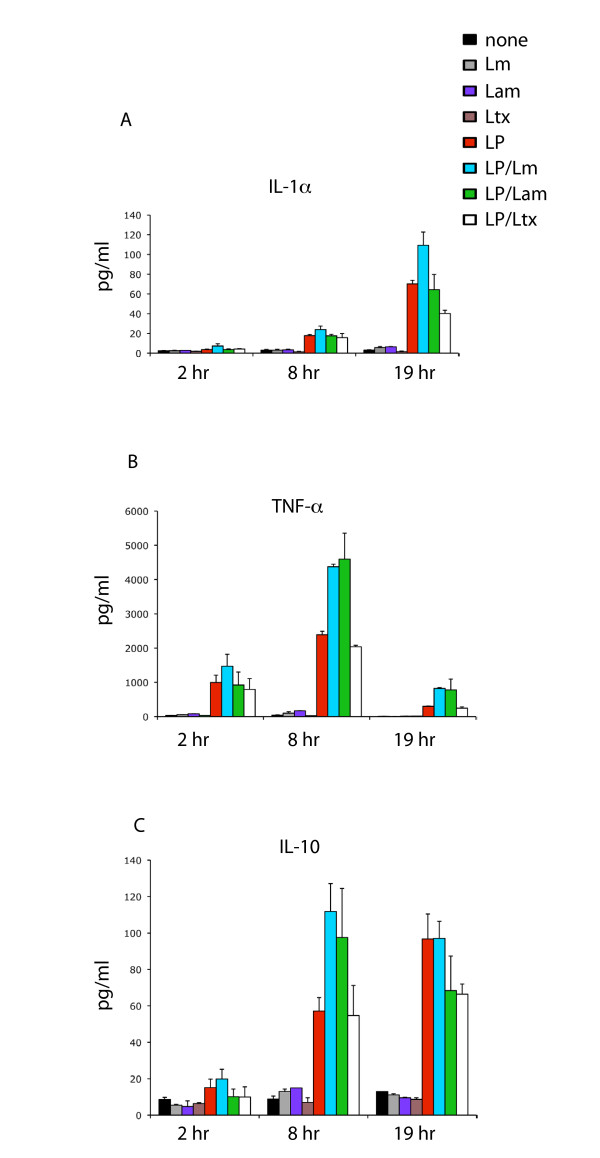
***Leishmania *augments release of IL-1α, TNFα and IL-10 following LPS induction**. Cells were incubated with 100 ng/ml LPS followed by *L. major*, *L. amazonensis *or latex beads at a parasite/bead: host cell ratio of 20:1 (indicated by blue, green and open bars, respectively). At 2 hr, 8 hr and 19 hr timepoints, culture supernatants were removed, centrifuged then assayed for cytokine expression by Bio-Plex assay as described in Methods. Media only, *L. major *alone, *L. amazonensis *alone, latex beads alone and LPS alone (black, gray, purple, brown and red bars, respectively) were used as controls. Data represented are averages of two independent replicate experiments, with error bars as denoted.

To determine if the suppressive effects of *Leishmania *reflected a generalized counter-inflammatory response against LPS-induced proinflammatory macrophage cytokines, we also analysed the effect of *Leishmania *uptake upon LPS-induction of the proinflammatory cytokines IL-1α and TNFα. As shown in Figure 3A, we found that, in contrast to its suppressive effects on IL-17 and IL-12, *L. major *augmented LPS-induced IL-1α 1.6-fold. Conversely *L. amazonensis *had no effect on LPS-induced IL-1α levels and latex beads showed a 1.7-fold suppressive effect. In contrast to IL-1α, both *L. major *and *L. amazonensis *augmented LPS-induced TNFα 1.8- and 1.9-fold, respectively at 8 hrs, the peak of the LPS induced TNFα response (Figure 3B). These data demonstrate that although *Leishmania *suppresses certain LPS-induced proinflammatory cytokines, it can simultaneously facilitate the production of other classical inflammatory cytokines. Interestingly, *L. amazonensis *generally showed a more suppressive effect than *L. major*.

We also investigated the impact of *Leishmania *uptake on LPS-induced IL-10, since this cytokine typically antagonizes the biological effects of classic proinflammatory cytokines such as IL-12. As shown in Figure 3C, at 8 hr *Leishmania *augmented LPS-induced IL-10, however by 19 hrs, the peak of the LPS-induced IL-10 response, the levels of IL-10 were not significantly different between LPS alone and both *Leishmania *species. In contrast, latex beads showed moderate suppression of LPS-induced IL-10 at this timepoint.

### Leishmania promotes the production of LPS-induced MIP-1α and MCP-1

**Figure 4:**
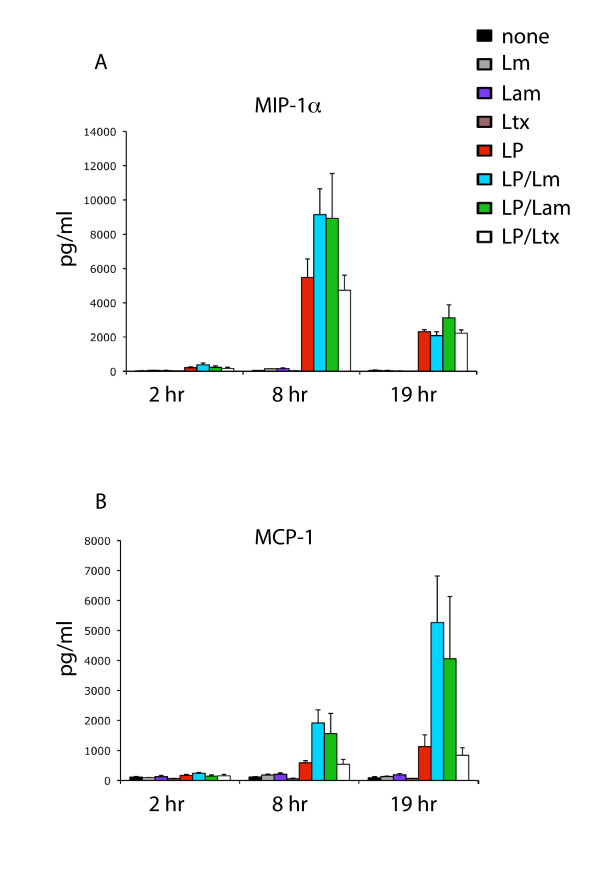
***Leishmania *promotes the production of LPS-induced MIP-1α and MCP-1**. Cells were incubated with 100 ng/ml LPS followed by the addition of *L. major*, *L. amazonensis *or latex beads at a parasite/bead: host cell ratio of 20:1 (indicated by blue, green and open bars, respectively). At 2 hr, 8 hr and 19 hr timepoints, 200 μl of culture supernatant was removed, centrifuged then assayed for cytokine expression by Bio-Plex assay as described in Methods. Media only, *L. major *alone, *L. amazonensis *alone, latex beads alone and LPS alone (black, gray, purple, brown and red bars, respectively) were used as controls. Data represented are averages of two independent replicate experiments, with error bars as indicated.

Since the chemokines macrophage inflammatory protein (MIP)-1α and macrophage chemoattractant protein (MCP)-1 have been shown to have an important role in limiting macrophage parasitic burden [24] we sought to determine whether *Leishmania *could suppress expression of these chemokines as a possible way to enhance parasitization. As shown in Figure 4A, at the peak of the LPS-induced response, *L. major *and *L. amazonensis *promoted LPS-induced MIP-1α 1.7-fold and 1.6-fold respectively, whereas latex beads had little effect. Analysis of LPS-induced MCP-1 showed that *L. major *and *L. amazonensis *dramatically augmented LPS-induced levels of this chemokine, with 4.7-fold and 3.6-fold upregulation respectively, whilst treatment of the LPS-induced macrophages with latex beads showed no significant impact on MCP-1 levels (Figure 4B). Both *L. major *and *L. amazonensis *were also found to suppress LPS-induced MIP-1β (data not shown).

**Figure 5:**
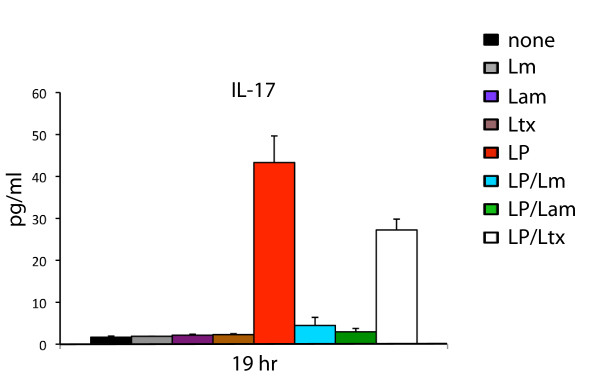
**ELISA analysis of Leishmania-mediated cytokine suppression**. Cells were incubated with LPS (100 ng/ml) followed by *L. major*, *L. amazonensis *or latex beads at a parasite/bead to host cell ratio of 20:1 (indicated by blue, green and open bars, respectively). At 19 hrs, culture supernatants were taken, centrifuged then assayed for IL-17 production by ELISA as described in Methods. Media only, *L. major *alone, *L. amazonensis *alone, latex beads alone and LPS alone (black, gray, purple, brown and red bars, respectively) were used as controls. Data represented are averages of two independent replicate experiments, with error bars as denoted.

Our findings were further validated by sampling cytokine levels using ELISA assays, as shown for IL-17 (Figure 5; data not shown).

## Discussion

Macrophages are key mediators of inflammatory responses that are important for host immune protection against infectious challenge. When such responses become dysregulated or chronically activated, however, they contribute to the development of inflammatory diseases such as atherosclerosis and arthritis. Although significant advances, such as the identification of TLRs and their associated intracellular signalling pathways, have contributed substantially to our understanding of how inflammatory responses are activated, critical gaps remain in our understanding of how some microbes avoid and even suppress host inflammatory responses. Previous studies have identified *Leishmania *as a eukaryote protozoan microbe that circumvents the classical host innate immune response, and also actively suppresses macrophage responses to strong inflammatory stimuli such as bacterial LPS [12,25-27]. These studies have typically been limited to analyses of a relatively low number of cytokines, and in general have not distinguished between the specific effects of *Leishmania *uptake and non-specific effects of generalized phagocytosis upon LPS-induced macrophage activation. We sought to further explore the modulatory effects of *Leishmania *upon LPS-induced macrophages by determining the specific effects of two clinically distinct species of *Leishmania *upon the kinetics of a broader repertoire of cytokine and chemokine responses elicited by LPS-activated macrophages.

Consistent with previous observations, our studies demonstrate that *Leishmania *efficiently evades robust activation of many cytokines and chemokines. In the context of LPS-induction, we found that the proinflammatory cytokines IL-17, IL-12 and IL-3, and the Th2 cytokines IL-4, IL-6 and IL-13 were all suppressed to varying degrees by both *L. major *and *L. amazonesis*. For each of these cytokines, the suppressive effects were more pronounced with *L. amazonensis *than *L. major*. Although we cannot rule out the possibility that the increased suppressive effects we observed for *L. amazonensis *were due to their marginally increased level of uptake compared to *L. major*, we feel it is unlikely that such a subtle increase in parasite uptake would lead to the striking increases in cytokine suppression we observed.

Although the latex bead controls were moderately suppressive at the peak of the LPS response, we found that in general, *Leishmania *were significantly more so. Another interesting finding of our study was that whilst *Leishmania *suppressed some LPS-induced responses, such as IL-4 and IL-13 at timepoints when the LPS response was maximal (usually 19 hrs), these responses were actually augmented by *Leishmania *at the 8 hr timepoint. This observation is reminiscent of the refractory period observed with *Toxoplasma gondii*-induced IL-12 production in dendritic cells [28], and may therefore not represent true suppression.

Although IL-17-producing Th17 cells have been the subject of intensive study recently and IL-17 production is critical for the expansion of innate immune cells, only one report has described the importance of macrophage-derived IL-17 in inflammatory disease [7]. Indeed, our study is the first to investigate the impact of *Leishmania *upon LPS-induced IL-17 production in macrophages. Our observations that both *L. major *and *L. amazonensis *promastigotes strongly and specifically suppress LPS-induced IL-17 production, provide a new model for studying mechanisms of IL-17 regulation.

Although we observed suppression of both IL-12p40 and IL-12p70, IL-12p40 suppression was more striking. Interestingly, our finding that *L. major *and latex beads had similar mildly suppressive effects suggests that phagocytic uptake alone contributes significantly to suppression of LPS-induced IL-12p70 during *L. major *infection. Our findings with *L. amazonensis *differ from those of Cameron *et al*., [12], which showed its close relative, *L. mexicana*, caused a more dramatic suppression of LPS-induced IL-12p70. Instead, our data are more comparable to previous findings with *L. major*-infected human monocytes [29]. These different observations may be attributable to distinct pathobiological properties known to exist even amongst closely related *Leishmania *species.

*L. major *but not *L. amazonensis *showed augmentation of LPS-induced IL-1α. Interestingly, IL-1α can promote Th1 responses that ameliorate disease progression in susceptible BALB/c [30] whilst Th1-biased C57BL/6 mice that are resistant to *L. major *remain susceptible to *L. amazonensis *[15]. Whether or not the differences in macrophage responses elicited by *L. amazonensis *versus *L. major *under our conditions contribute to the increased severity of disease typically associated with *L. amazonensis *compared to *L. major*, remains to be determined.

Production of IL-10 by *Leishmania *alone was not strikingly increased above controls, however at 8 hrs post treatment, both *Leishmania *species significantly augmented LPS-induced levels of IL-10. Since IL-10 suppresses production of inflammatory cytokines, such as IL-12, it is interesting that a classical proinflammatory stimulus such as LPS also induces IL-10. Indeed, these data are consistent with recent findings [31] and may reflect IL-10-mediated negative feedback regulation. Since IL-10 is a strong suppressor of host cytokine production and promotes disease progression in leishmaniasis [32,33], it is possible that the cytokine-suppressive effects we observed could be attributable to *Leishmania*-augmented IL-10 production. We feel this is unlikely however, since although IL-10 is a potent suppressor of macrophage TNF-α [34], the increased levels of IL-10 released by LPS/*Leishmania *in our study did not prevent augmentation of TNFα production. Furthermore, in contrast to both *L. major *and *L. mexicana*, absence of IL-10 does not protect against *L. amazonensis *infection [35], suggesting that *L. amazonensis *employs IL-10-independent immunomodulatory mechanisms.

Although these studies provide new insight into the regulation of macrophage inflammatory responses by *Leishmania*, it should be emphasized that they were performed *in vitro *using peritoneal macrophages to identify novel mechanisms of macrophage inflammatory modulation. How these *in vitro *findings translate to *in vivo *models of *Leishmania *infection has not yet been clarified. It is possible, for example, that peritoneal macrophages may behave differently to the inflammatory macrophages found at infection sites in the skin during *Leishmania *inoculation. Further, the metacyclic-enriched promastigote population inoculated during natural infection may have different effects upon macrophage responses than the less-defined heterogenous population of stationary phase parasites used in our studies. It is also important to emphasize that *Leishmania *amastigote stages may elicit different macrophage responses than promastigotes. As such, future studies with purified metacylic organisms and distinct macrophage populations will be helpful in determining the extent to which our novel findings pertain to *Leishmania *infection *in vivo*.

We also emphasize that studies of promastigote-mediated modulation of LPS-induced cytokine responses may have limited relevance in the context of natural *Leishmania *infections, since *Leishmania *possesses no known TLR4 agonists and there are no data confirming that TLR4 agonists are introduced into the sandfly bite site. Instead, however, these studies may be relevant for the design of protective adjuvant-based vaccination strategies against *Leishmania*, using TLR agonists to promote optimal inflammatory cytokine profiles that may facilitate sustained Th1 responses required for protective immunity against this pathogen.

## Conclusions

We have explored the cytokine-modulatory effects of two clinically distinct species of *Leishmania *upon the kinetics of a range of LPS-induced macrophage inflammatory responses. We find both suppression and promotion of LPS-induced responses, indicating the selective suppression and augmentation of specific cytokine induction mechanisms. Our studies provide foundations to pursue functional studies to further elucidate the molecular mechanisms that underlie the distinct cytokine responses to *Leishmania *that we observed. Such investigations of *Leishmania*-mediated modulation of host cytokine responses will advance our understanding of inflammatory responses and likely promote new avenues for therapeutic intervention against inflammatory diseases.

## Abbreviations

TLR: Toll-like receptor; NF-κB: nuclear factor kappa-light-chain enhancer of activated B cells; CR3: complement receptor 3; IL-: interleukin; MIP-: macrophage inflammatory protein; MCP-1: macrophage chemotactic protein-1.

## Competing interests

The authors declare that they have no competing interests.

## Authors' contributions

NJL executed all experiments and assisted BLK in the experimental design. BLK was responsible for the experimental design and writing the manuscript. Both Authors have read and approved the final manuscript.
